# ICTV Virus Taxonomy Profile: *Pseudoviridae*

**DOI:** 10.1099/jgv.0.001563

**Published:** 2021-02-02

**Authors:** Carlos Llorens, Beatriz Soriano, Mart Krupovic, ICTV Report Consortium

**Affiliations:** ^1^​ Biotechvana, Scientific Park University of Valencia, 46980, Paterna, Valencia, Spain; ^2^​ Archaeal Virology Unit, Institut Pasteur, 75015 Paris, France

**Keywords:** *Pseudoviridae*, ICTV Report, taxonomy

## Abstract

*Pseudoviridae* is a family of reverse-transcribing viruses with long terminal repeats (LTRs) belonging to the order *Ortervirales*. Pseudoviruses are commonly found integrated in the genomes of diverse plants, fungi and animals and are broadly known as Ty1/Copia LTR retrotransposons. Inside the cell, they form icosahedral virus particles, but unlike most other viruses, do not have an extracellular phase. This is a summary of the ICTV Report on the family *Pseudoviridae*, which is available at ictv.global/report/pseudoviridae.

## Virion

**Fig. 1.**
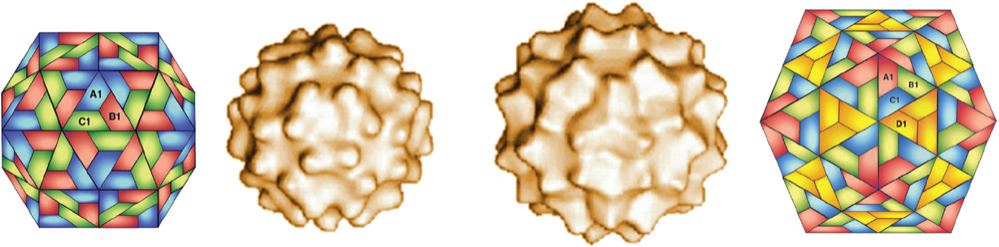
Saccharomyces cerevisiae Ty1 virus particles formed from truncated capsid protein (aa 1–381). The surface structures of two forms (*T*=3, left; *T*=4, right) of around 30–40 nm determined by cryo-electron microscopy, are flanked by the corresponding schematic models. (Courtesy of H. Saibil, adapted from [3] with permission from American Society for Microbiology.)

Pseudoviruses form intracellular, somewhat irregularly shaped virus-like particles (VLPs) 30–40 nm in diameter, which do not display infectivity and remain intracellular (Table 1). VLPs are formed by self-assembly of proteins encoded by the *gag* gene, namely the capsid (CP) and nucleocapsid (NC) proteins, which are homologous to the equivalent proteins of retroviruses and other members of the order *Ortervirales* [1]. Expression of truncated Gag protein variants yields icosahedral VLPs of different diameters but with a mean radius of 20 nm built on the *T*=3 or *T*=4 lattice (Fig. 1) [2, 3].

**Table 1.** Characteristics of members of the family *Pseudoviridae*

Example:	Saccharomyces cerevisiae Ty1 virus (M18706), species *Saccharomyces cerevisiae Ty1 virus*, genus *Pseudovirus*
Virion	Virions are icosahedral (*T*=3 or 4) and might be enveloped
Genome	Two identical copies of linear single-stranded RNA
Replication	Replication by reverse transcription primed with a host-encoded tRNA
Translation	Genomic RNA is translated into one or more polyproteins
Host range	Fungi, plants and animals
Taxonomy	Realm *Riboviria*, kingdom *Pararnavirae*, phylum *Artverviricota*, class *Revtraviricetes*, order *Ortervirales*, family *Pseudoviridae*; the genera *Pseudovirus, Hemivirus* and *Sirevirus* include >30 species

## Genome

**Fig. 2.**
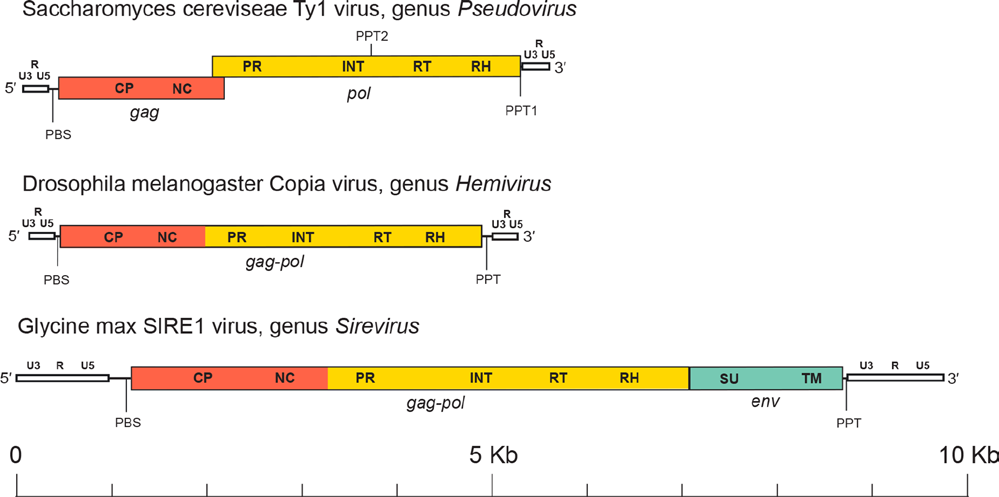
Pseudovirid genome organization. LTRs are white and show labels for the U3, R and U5 regions. Other labels are: capsid (CP); integrase (INT); long terminal repeat (LTR); nucleocapsid (NC); polypurine tract (PPT); primer binding site (PBS); protease (PR); reverse transcriptase (RT); ribonuclease H (RH); surface (SU); transmembrane (TM).

The genome of pseudovirids ranges from 4 kb to >9 kb and has an internal region flanked by two identical non-coding sequences called long terminal repeats (LTRs) (Fig. 2). LTRs are variable in size and contain three regions, named U3-R-U5 in analogy to retroviral LTRs. U3 contains promoters, R is repeated on each end of the transcript, and U5 constitutes the first portion of the reverse-transcribed genome. The internal region is delimited by two short motifs: the primer binding site (PBS), which is located downstream of the 5′-LTR and is usually complementary to the initiator tRNA^Met^, and the polypurine tract (PPT), which is upstream of the 3′-LTR. The internal region may contain one (*gag-pol*), two (*gag* and *pol*) or three (*gag*, *pol* and *env*) ORFs. The Gag polyprotein includes domains for the CP and NC proteins, while Pol includes domains for the protease (PR), integrase (INT) and reverse transcriptase-ribonuclease H (RT-RH). Members of the genus *Sirevirus* carry a third ORF downstream of *gag*-*pol* encoding a putative envelope protein [4].

## Replication

The replication of pseudovirids resembles that of retroviruses and occurs via reverse transcription in the VLP. The cellular tRNA molecule, typically initiator tRNA^Met^, which is packaged into the VLP, anneals to the viral RNA genome at the PBS complementary to the 3′-end of that tRNA and is used by RT as a primer to start reverse transcription. Once the full-length proviral cDNA is synthetized, it is imported into the nucleus, where it is integrated into a chromosomal target site by INT. The integrated form (equivalent to the retroviral provirus) is transcribed by the host RNA polymerase II to generate a new viral RNA molecule, which is translated to produce the Gag and Pol polyproteins and is packaged into the VLPs to reinitiate the replication cycle.

## Taxonomy

Current taxonomy: ictv.global/taxonomy. The family *Pseudoviridae* belongs to the order *Ortervirales* [5] and includes the genera *Pseudovirus*, *Hemivirus* and *Sirevirus*. Current classification is based on host tropism, gene content and the length of the tail of the tRNA used as a primer to initiate reverse transcription. Hemiviruses differ from members of the genus *Pseudovirus* in that they use only a short segment of the tRNA. By contrast, sireviruses are restricted to plants and mostly encode a protein equivalent to retroviral Env downstream of the *gag*-*pol* gene [4]. Further updates and revisions in the genus demarcation of pseudovirids are expected to be based on phylogenetic criteria as described in the full ICTV Report (see Resources).

## Resources

Full ICTV Report on the family *Pseudoviridae*: ictv.global/report/pseudoviridae.

Gypsy Database (GyDB) devoted to viruses and mobile genetic elements: http://gydb.org.
